# Safety and Effectiveness of Isavuconazole Treatment for Fungal Infections in Solid Organ Transplant Recipients (ISASOT Study)

**DOI:** 10.1128/spectrum.01784-21

**Published:** 2022-02-16

**Authors:** Arnau Monforte, Ibai Los-Arcos, Maria Teresa Martín-Gómez, David Campany-Herrero, Judith Sacanell, Cristina Berastegui, Ester Márquez-Algaba, Abiu Sempere, Xavier Nuvials, Maria Deu, Lluís Castells, Francesc Moreso, Carles Bravo, Joan Gavaldà, Oscar Len

**Affiliations:** a Department of Infectious Diseases, Hospital Universitari Vall d’Hebron, Barcelona, Spain; b Department of Medicine, Universitat Autònoma de Barcelona, Barcelona, Spain; c Department of Microbiology, Hospital Universitari Vall d’Hebron, Barcelona, Spain; d Department of Pharmacy, Hospital Universitari Vall d’Hebron, Barcelona, Spain; e Intensive Care Unit, Hospital Universitari Vall d’Hebron, Barcelona, Spain; f Department of Pneumology, Hospital Universitari Vall d’Hebron, Barcelona, Spain; g Department of Thoracic Surgery, Hospital Universitari Vall d’Hebron, Barcelona, Spain; h Liver Unit, Hospital Universitari Vall d’Hebron, Barcelona, Spain; i Centro de Investigación Biomédica de Enfermedades Hepáticas y Digestivas (CIBERehd), Instituto de Salud Carlos III, Madrid, Spain; j Department of Nephrology, Hospital Universitari Vall d’Hebron, Barcelona, Spain; University of Texas Southwestern Medical Center

**Keywords:** isavuconazole, fungal infection treatment, solid organ transplantation

## Abstract

Isavuconazole (ISA) is an alternative treatment for Aspergillus spp. and other fungal infections, but evidence regarding its use in solid organ transplant recipients (SOTR) is scarce. All SOTR who received ISA for treatment of a fungal infection (FI) at our center from December 2017 to January 2021 were included. The duration of the treatment depended on the type of infection. All patients were followed up to 3 months after treatment. Fifty-three SOTR were included, and the majority (44, 83%) were lung transplant recipients. The most frequently treated FI was tracheobronchitis (25, 46.3%). Aspergillus spp. (43, 81.1%); specially A. flavus (16, 37.2%) and A. fumigatus (12, 27.9%), was the most frequent etiology. Other filamentous fungi including one mucormycosis, and four yeast infections were treated. The median duration of treatment was 81 days (IQR 15-197). Mild gamma-glutamyltransferase elevation was the most frequent adverse event (34%). ISA was prematurely discontinued in six patients (11.3%) due to mild hepatotoxicity (2), fatigue (2), gastrointestinal intolerance (1) and myopathy (1). The mean tacrolimus dose decrease was 30% after starting ISA. Seven patients received ISA with mTOR inhibitors with good tolerability. Two patients developed breakthrough FI (3.8%). Among patients who completed the treatment, 27 (50.9%) showed clinical cure and 15 (34.1%) presented fungal persistence. Three patients (6%) died while on ISA due to FI. ISA was well tolerated and appeared to be an effective treatment for FI in SOTR.

**IMPORTANCE** We describe 53 solid organ transplant recipients treated with isavuconazole for fungal infections. Because its use in clinical practice, there is scarce data of its use in solid organ transplant recipients, where interactions with calcineurin inhibitors and mTOR and adverse drug events have limited the use of other triazoles. To the best of our knowledge, this is the first article describing the safety regarding adverse events and drug interactions of isavuconazole for the treatment of fungal infections in a cohort of solid organ transplant recipients. Also, although this is a noncomparative study, we report some real world effectivity data of these patients, including treatment of non-Aspergillus fungal infections.

## INTRODUCTION

Solid organ transplant recipients (SOTR) have a significant risk of invasive infection caused by fungi such as Aspergillus spp. Depending on the SOTR modality, the overall incidence of invasive aspergillosis (IA) ranges between 0.1% up to 15% (1, 2), making it the most common cause of fungal infection (FI) in lung transplant recipients (3). A previous study conducted in our center showed a 5% incidence of IA in a cohort of 412 lung transplant recipients (4). The mortality rate of aspergillosis in lung transplant recipients remains very high, reaching 25% in patients affected with ulcerative tracheobronchitis, and 67–82% in patients developing invasive lung disease (1).

Voriconazole is recommended as the first line drug for the treatment of invasive aspergillosis (5, 6). However, its use in SOTR is limited by interactions and toxicity issues. The strong inhibition it exerts over the CYP3A4 increases the levels of immunosuppressive drugs such as tacrolimus or cyclosporine, posing a great risk of developing severe side effects such as renal impairment. The concomitant administration of voriconazole with sirolimus is contraindicated due to serious drug interactions (7). Moreover, adverse events such as hepatotoxicity, visual disturbances, phototoxicity and increased risk of squamous cell carcinoma are frequently seen in these patients (8, 9).

Isavuconazole is a new extended-spectrum triazole that has been approved for the treatment of invasive aspergillosis and mucormycosis, with fewer drug-related adverse events (10). However, there is little data on the use of isavuconazole for treatment of FI in SOTR. The objective of this study was to assess the suitability, safety, and effectiveness of isavuconazole in solid organ transplantation in daily clinical practice.

## RESULTS

**FIG 1:**
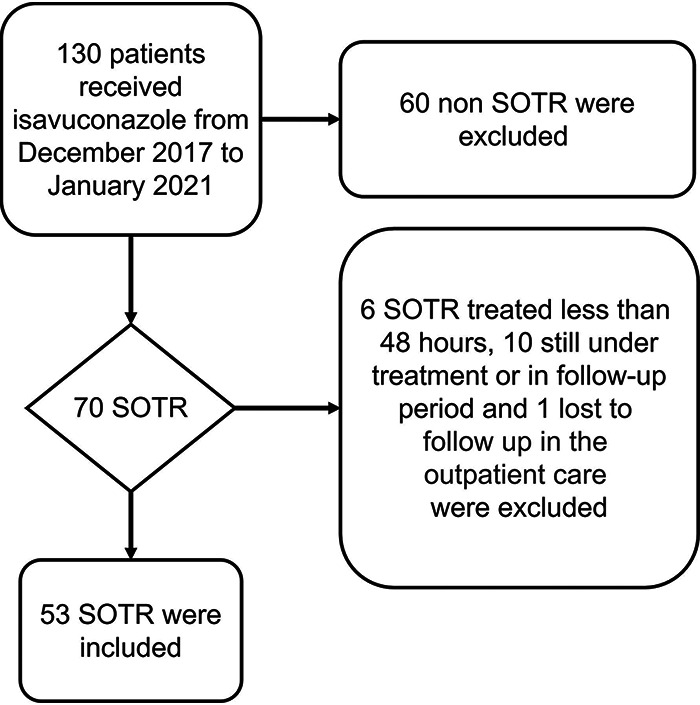
Flow chart. SOTR, solid organ transplant recipient.

One hundred and thirty patients received isavuconazole from December 2017 until January 2021. Fifty-three of them were SOTR meeting the inclusion criteria for the study (Fig. 1).

The median age was 60 years old (IQR 46–65), and 37.7% were female. Forty-four (83%) were lung transplant recipients, with pulmonary fibrosis being the most frequent chronic respiratory disease that led to transplant. Four (7.5%) were liver transplant recipients and four (7.5%) were kidney transplant recipients, with one (1.9%) combined liver-kidney transplant. Antifungal prophylaxis with nebulized liposomal amphotericin B was used in 43 lung transplant recipients. Most of SOTR (50, 94.3%) received triple immunosuppression treatment initially, based on calcineurin inhibitors (tacrolimus in all cases), mycophenolate mofetil and corticosteroids. Main baseline and transplant characteristics are described in Table 1.

**TABLE 1:** Baseline and transplant characteristics^*a*^

Baseline and transplant characteristics	All cases (*n* = 53)
Median (range) age (yr)	60 (46-65)
Female patients	20 (37.7)
Liver transplant recipients	4 (7.5)
Kidney transplant recipients	4 (7.5)
Combined liver-kidney transplant recipient	1 (1.9)
Lung transplant recipients	44 (83)
Respiratory disease	
Pulmonary fibrosis (IPF, connective tissue disease, hypersensibility pneumonitis…)	24 (54.5)
COPD	9 (20.5)
Pulmonary hypertension	6 (13.6)
Cystic fibrosis	4 (9.1)
Previous lung transplant	2 (4.5)
Bilateral lung transplant	30 (68.2)
Bronchial stenosis	10 (22.7)
Bronchial dilatations	5 (11.4)
Bronchial stent	4 (9.1)
CLAD	10 (22.7)
BOS	6 (60)
RAS	2 (20)
Mixed or undefined CLAD	2 (20)
Emergency transplant procedure	6 (11.3)
ECMO pretransplant	1 (1.9)
ECMO in early post transplant	2 (3.8)
Use of ECC	14 (26.4)
Previous FI	11 (20.8)
Aspergillus *spp.*	8 (72.7)
Immunosuppression with tacrolimus, mycophenolate mofetil and corticosteroids	50 (94.3)
Antifungal prophylaxis	45 (84.9)
Nebulized amphotericin B	43 (95.6)
Micafungin	1 (2.2)
Under antifungal treatment due to FI before transplantation	1 (2.2)

a Data are expressed as numbers (%) unless otherwise indicated. BOS, bronchiolitis obliterans syndrome; CLAD, chronic lung allograft dysfunction; COPD, chronic obstructive pulmonary disease; ECC, extracorporeal circulation; ECMO, extracorporeal membrane oxygenation; FI, fungal infection; ICU, intensive care unit; IQR, interquartile range; IPF, idiopathic pulmonary fibrosis; RAS, restrictive allograft syndrome.

The most frequent FI was tracheobronchitis (25, 46.3%), followed by fungal pneumonia (7, 13%). According to definitions, 44 (81.5%) were probable FI, 4 (7.4%) were proven FI and 6 (11.1%) were not proven or probable FI. An invasive form of FI was diagnosed in 46.3% of them. Median time from transplantation to FI was 5.7 months (IQR 2–40). All data regarding infection and fungal isolates is displayed in Table 2. Fifty-three different fungal isolates infected SOTR, of which 43 (82.7%) were Aspergillus spp. A. flavus *complex* 16 (37.2%) and A. fumigatus 12 (27.9%) accounted for the majority of FI in this SOTR cohort. There were four yeast infections (2 *Trichosporon ashaii*, 1 Candida albicans and 1 Saccharomyces cerevisiae). Isavuconazole MIC was equal or inferior to 0.5 μg/mL in all Aspergillus spp. isolates, and greater than 1 μg/mL in only two molds (*Lomentospora prolificans* and Alternaria alternata).

**TABLE 2:** Fungal infection and treatment^*a*^

Fungal infection	Description
Median (range) time (mo) from transplant to infection	5.7 (1.7–40.2)
Identified fungal species	53 species in 44 SOTR
Aspergillus *spp*	43 (81.1)
Alternaria alternata	1 (1.9)
* Diaporthe spp.*	1 (1.9)
* Lomentospora prolificans*	1 (1.9)
* Purpureocillium lilacinus*	1 (1.9)
Unidentified new mold species (28)	1 (1.9)
Mucormycosis	1 (1.9)
* Trichosporon asahii*	2 (3.8)
Candida albicans	1 (1.9)
Saccharomyces cerevisiae	1 (1.9)
No fungal isolate	9 SOTR
Type of FI^*b*^	54 infections in 53 SOTR^*c*^
Tracheobronchitis	25 (46.3)
Fungal pneumonia	7 (13)
Bronchial anastomotic infection	2 (3.7)
Other	
Micetoma	6 (11.1)
Cutaneous infection	3 (5.6)
Disseminated FI	2 (3.7)
Osteomyelitis	1 (1.9)
Chronic otitis media	1 (1.9)
Isolation in donor	1 (1.9)
No proven or probable FI	6 (11.1)
Previous antifungal treatments	17 (32.1)
Voriconazole	7 (13.2)
Nebulized amphotericin B	5 (9.4)
Systemic echinocandin (micafungin or anidulafungin)	5 (9.4)
Duration of previous treatment (days). Mean (IQR)	13 (4–37)
Reason to stop previous treatment (*n* = 17)	
Intravenous-to-oral switch and avoiding interactions	6 (35.3)
No previous clinical benefit	5 (29.4)
Switch according to antifungal susceptibility	3 (17.6)
Adverse events with previous treatment	3 (17.6)

a Data are expressed as numbers (%) unless otherwise indicated. EORTC/MSGERC, European Organization for Research and Treatment of Cancer and the Mycoses Study Group Education and Research Consortium; FI, fungal infection; IQR, interquartile range; ISA, isavuconazole; ISHLT, International Society for Heart and Lung Transplantation; SOTR, solid organ transplant recipient.

b All cases were definite or probable fungal infection according to ISHLT and EORTC/MSGERC criteria except when indicated.

c One patient was treated for fungal tracheobronchitis and subcutaneous infection at the same time.

Among all patients treated, 27 (50.9%) showed clinical cure at EOT (44.4% for invasive and 55.6% for noninvasive FI). Culture conversion was confirmed in 17 out of 44 SOTR with an initial fungal isolation (38.6%). Cultures were not performed at EOT in 12 (27.3%) of them. In lung transplant recipients, 13 of 22 with tracheobronchitis (59.1%) presented clinical cure at EOT and 9 of 19 (47.4%) presented culture conversion. Fifteen out of 53 SOTR (28.3%) died while on treatment with isavuconazole, and all-cause mortality within 3 months was 24 (45.3%). Mortality was related to fungal infection in only 3 cases (6%), excluding localized cutaneous and subcutaneous diseases: two cases of fungal pneumonia and one of disseminated aspergillosis with pulmonary and central nervous system involvement. All information is summarized in Table 3.

**TABLE 3:** Clinical cure, mortality and culture conversion^*a*^

Clinical cure, mortality and culture conversion	Description
Clinical cure at EOT (*n* = 53)	27 (50.9)
Culture conversion in patients with fungal species isolation (*n* = 44)	
Culture conversion at EOT	17 (38.6)
Fungal persistence at EOT	15 (34.1)
Cultures were not performed at EOT	12 (27.3)
Deceased patients at EOT (*n* = 53)	
Deceased patients at EOT	15 (28.3)
Deceased patients at 90 days after EOT	24 (45.3)
Bacterial infection	7 (30.4)
CLAD	5 (20.8)
SARS-CoV-2 infection	4 (16.7)
Fungal infection	3 (12.5)
Relapse of pretransplant disease	2 (8.3)
Hypovolemic shock	2 (8.3)
Lung neoplasm	1 (4.2)
Patients deceased in ICU	14 (58.3)

a Data are expressed as numbers (%) unless otherwise indicated. CLAD, chronic lung allograft disease; EOT, end of treatment; ICU, intensive care unit.

Isavuconazole was initiated as a targeted treatment in 44 episodes (83%) or empirically in 9 SOTR (17%). It was started after a previous antifungal treatment in 17 episodes (32.1%), with voriconazole being the most frequently discontinued treatment. The main reason to switch treatment was intravenous-to-oral switch while avoiding interactions in six cases (33.3%). Most of the treatments started during hospital admission (43, 81.1%), 24 started in the conventional ward, and 19 in the intensive care unit (ICU). 45.2% of SOTR were administered isavuconazole intravenously, and 15% were switched to oral during the treatment. The median duration of treatment with isavuconazole was 81 days (IQR 15–197). Two patients (3.8%) developed breakthrough FI: Aspergillus fumigatus with confirmed susceptibility to azoles, and *Scopulariopsis* sp. with isavuconazole MIC >8 μg/mL.

**FIG 2:**
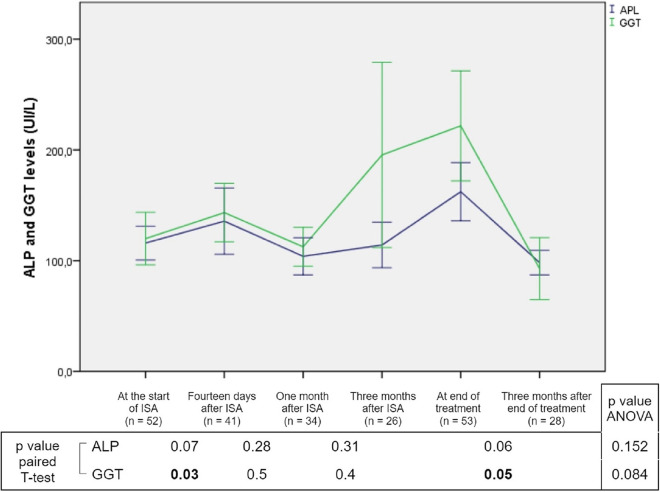
Change in liver cholestatic enzymes during isavuconazole treatment. ALP, alkaline phosphatase; GGT, gamma-glutamyltransferase; ISA, isavuconazole; SOTR, solid organ transplant recipient. Bolded values are *P* ≤ 0.05.

Twenty-six (49.1%) presented at least one adverse event. Elevation of liver enzymes was detected in 18 (34%) SOTR, all of them graded as G1. It predominantly accounted for alkaline phosphatase (ALP) and gamma-glutamyl transferase elevation. Paired sample tests did not find any significant changes in ALP, although gamma-glutamyl transferase values significantly increased after 14 days of isavuconazole and decreased after 3 months of its discontinuation. The multivariate analysis did not find any significant changes during treatment (data shown in Fig. 2). No bilirubin elevation or liver failure was observed. Myopathy was reported in 7 (13.2%) SOTR, with 6 relating to a concomitant use of corticosteroids. All adverse events were graded G1 or G2, none of them being severe or medically significant. Isavuconazole was prematurely discontinued in 6 SOTR (11.3%): two for hepatotoxicity G1, two due to fatigue G1, one due to digestive intolerance G2, and one due to myopathy G2. All adverse events are described in Table 4.

**TABLE 4:** Adverse events during isavuconazole treatment^*a*^

Adverse event 26 SOTR (49.1%)	All SOTR (*n* = 53)	Description	CTCAE
Cholestatic liver enzymes elevation	18 (34)	13 (72.2) presented ALP/GGT elevation	18 G1
		5 (27.7) presented GGT elevation alone	
Myopathy	7 (13.2)	Six associated with concomitant corticosteroid. One discontinued ISA	5 G1 2 G2
Gastrointestinal symptomatology	3 (5.7)	Nausea and vomiting, one discontinued ISA	2 G1 1 G2
Neurological disorders	2 (3.8)	One presented dizziness and vertigo One presented eye floaters	2 G1
Hepatotoxicity	2 (3.8)	AST/ALT and ALP/GGT elevation. Both discontinued ISA	2 G1
General disorders	3 (5.7)	Fatigue, two of them discontinued ISA	3 G1
	1 (1.9)	Wt loss	1 G1
Total of premature discontinuation of ISA	6 (11.3%)		4 G1 2 G2

a Data are expressed as numbers (%) unless otherwise indicated. ALP, alkaline phosphatase; ALT, alanine aminotransferase; AST, aspartate aminotransferase; CTCAE, Common Terminology Criteria for Adverse Events; GGT, gamma-glutamyltransferase; ISA, isavuconazole; SOTR, solid organ transplant recipient.

The dose of tacrolimus was changed during the first 14 days of treatment in 40 (85.1%) SOTR. A median decrease of 2 mg was observed over this period (a 30% mean dose decrease). After discontinuation of isavuconazole, tacrolimus dose was increased in 24 (50%) SOTR within 14 days, with a median dose increment of 1 mg (a 20% mean dose increase). Seven SOTR (13.2%) received mTOR inhibitors during isavuconazole treatment (sirolimus in six and everolimus in one of them), with overall good tolerance. The dose of mTOR inhibitors needed to be adjusted within the first 14 days, both after introduction (6, 85.7%) and discontinuation (2, 40%) of isavuconazole. Finally, labile INR values were reported in one patient during treatment with isavuconazole and acenocoumarol, which could be adjusted without discontinuation of any medication. All information is shown in Table 5.

**TABLE 5:** Frequency of immunosuppressive treatment and dose changes after introduction and discontinuation of isavuconazole^*a*^

Immunosuppressive	On isavuconazole	Off isavuconazole
Tacrolimus	*n* = 47	*n* = 48
Initial dose adjustment	22 (46.8) decreased dose	17 (35.4) increased dose 2 (4.1) decreased dose
Adjustment in first 14 days	40 (85.1)	24 (50)
mTOR inhibitor Sirolimus Everolimus	*n* = 7 6 (85.7) 1 (14.3)	*n* = 5 4 (80) 1 (20)
Adjustment in first 14 days	6 (85.7) decreased dose	2 (40) increased dose

a Data are expressed as numbers (%) unless otherwise indicated.

## DISCUSSION

In this non-comparative prospective observational study, treatment with isavuconazole was found to be well tolerated and effective in SOTR. Most of the cases were lung transplants recipients, who have a higher risk of filamentous FI than kidney or liver transplant recipients (1, 11), which lead to its overrepresentation in this study. Despite the fact that most isolated species were susceptible to isavuconazole, some patients did not present clinical improvement or culture conversion. Most SOTR suffering FI were treated during hospitalization (one third admitted in ICU) and had severe conditions, which probably had an impact on cure rates and culture conversion. A high mortality was expected in FI according to previous reports (12, 13), although most of them were unrelated to FI. Finally, infections due to non-Aspergillus molds in immunocompromised patients are difficult to treat and have high mortality rates (14).

The role of isavuconazole in FI has been evaluated in several studies, although few of them include a significant population of solid organ transplant recipients (15). In the phase III clinical trial (SECURE), isavuconazole was not inferior to voriconazole in the treatment of invasive disease due to filamentous fungi (10). Besides, isavuconazole presented fewer adverse effects such as hepatobiliary, ocular disorders and skin disorders. The main population of the study included patients diagnosed with hematological neoplasm and none of them was a SOTR. In the open VITAL trial, only one patient in the primary treatment group of isavuconazole for mucormycosis was a SOTR (16). Another retrospective study assessing isavuconazole tolerability in prophylaxis included 144 lung transplant recipients who received this azole. They concluded a lesser need to discontinue isavuconazole compared to voriconazole due to adverse events (12). There are previous reports of good tolerance of prolonged isavuconazole course in prophylaxis (17), but to the best of our knowledge, this is the first article describing its use for the treatment of FI in SOTR. In our cohort, patients who had adverse reactions with voriconazole (3) could complete more days of treatment with isavuconazole. One patient who presented hepatotoxicity with voriconazole also presented hepatotoxicity with isavuconazole after 392 days of treatment, although the FI was clinically cured and achieved culture conversion at that time, so no other antifungal treatment was prescribed. Another patient did not tolerate both azoles due to myopathy, fatigue and anorexia. Adverse events were not negligible in our ailing patient population, although very few led to discontinuation of treatment. Cholestatic liver enzyme elevation was the most frequently reported adverse event, despite the initial median values being above the normal values in this SOTR cohort (other medication or pathologies such as bacterial infection could probably be involved). Nonetheless, there were no significant differences at the start and after discontinuation of isavuconazole and no clinical impact was noted. Other conditions probably favored some adverse events reported in real clinical practice, such as the use of corticosteroids in apparition of myopathy. On the other hand, and unlike other last generation azoles, isavuconazole can be administered intravenously in case of renal failure due to the lack of cyclodextrin as an excipient. This condition favors its use from the beginning and its sequencing to the oral form.

Isavuconazole appears to be a moderate inhibitor of CYP3A4 with an increase of 1.8- and 2.3-fold in the area under the curve of plasma concentration of sirolimus and tacrolimus (18–20). One study analyzed isavuconazole as a universal prophylaxis in 55 SOTR (eight lung transplant recipients) (21). They concluded that after the suspension of isavuconazole, the concentration of tacrolimus was reduced, with the most important reduction observed in liver transplant recipients. Similarly, the tacrolimus dose was reduced in our patients upon initiation of isavuconazole and increased after discontinuation to maintain adequate plasmatic concentrations. The doses of immunosuppression were manageable in real clinical practice according to the plasmatic level measurement during the treatment. However, there is a high interindividual variability which prevents a sole recommendation in dose variation. Concomitant treatment with mTOR inhibitors and azoles was also described in this study, showing steady plasmatic levels during the treatment and no related adverse events. This is a very important issue since the concomitant use of mTOR inhibitors and voriconazole is contraindicated (18).

Although effectiveness was not the main objective of this study, clinical cure rates and mortality were similar to the ones reported in the SECURE trial (10). Even though culture conversion was also reported, cultures were not performed in 27.3% of SOTR. Nonetheless, the absence of a comparator group in real world conditions, the baseline differences of our patients and different presenting features and courses of FI make these results non comparable and should be interpreted with caution. Some non-Aspergillus FI were treated with success: an empyema which led to fungemia by *Trichosporon asahii*, two tracheobronchitis due to *Lomentospora prolificans* and *Purpureocillium lilacinus*, or a cutaneous infection by Alternaria alternata along with surgical debridement. Other diseases such as pulmonary mucormycosis were treated with initial success, as culture conversion was achieved, although the patient died due to bacterial sepsis. Despite the limited data, this experience supports the use of isavuconazole in difficult-to-treat non-Aspergillus infections in this population.

Finally, there are reports of breakthrough FI during treatment or prophylaxis with isavuconazole in SOTR, which ranged between 3% to 10% (12, 22). Similarly, breakthrough FI were noted in two patients (3.8%) in our cohort, with one of them susceptible to azoles. Although sub-therapeutic dosing could be suspected, plasmatic levels are not measured in clinical practice, as most studies performed showed limited pharmacokinetic variability even in SOTR (19).

This study has some limitations. First, it is an observational single center study without a comparator group. Second, the heterogeneity of types of infection and fungal isolates with a limited sample size. All in all, these results are extracted from real life clinical practice and could probably be generalized to other clinical settings in the treatment of FI in SOTR.

In conclusion, ISA was well tolerated and appeared to be an effective treatment for FI in SOTR. Immunosuppressive drug interactions were correctly managed in daily clinical practice including patients taking concomitant mTOR inhibitors. Further studies should clarify more issues in the treatment of FI with isavuconazole in SOTR.

## MATERIALS AND METHODS

### Patients and setting.

A non-comparative prospective observational study conducted at Hospital Universitari Vall d’Hebron in Barcelona was performed on all liver, kidney or lung transplant adult recipients treated with isavuconazole from December 2017 to January 2021.

We included all consecutive patients over 18 years old who accomplished more than 48 h of treatment for any FI. No fungal colonizations were included, as they were not treated in our patients. We excluded SOTR with a life expectancy of less than 72 h. As per protocol, all patients undergoing lung transplant in our center receive 25 mg (6 mL) of nebulized liposomal amphotericin B (n-LAB) three times per week for the first 60 days, 25 mg once weekly between 60 and 180 days, and 25 mg once every 2 weeks thereafter. Fungal cultures are performed in bronchoaspirate before lung transplant in the donor, the recipient and the organ preservation fluid. Systematic surveillance bronchoscopy after lung transplant is performed prior to discharge. It routinely includes transbronchial lung biopsy, fungal culture and galactomannan antigen measurement. Further bronchoscopies are performed at the discretion of the attending physician when patients have impaired forced ventilatory volume (FEV1) or clinical signs of infection. Echinocandin-based prophylaxis was restricted to high-risk liver transplant recipients (1), and no renal transplant recipients received antifungal prophylaxis. Isavuconazole was prescribed as oral or intravenous treatment with a loading dose of 200 mg every 8 h for the first 48 h and thereafter with 200 mg every 24 h, without therapeutic drug monitoring.

### Data collection.

A dedicated database was used to enter all data since the first day of treatment. Patients were followed for 90 days (3 months) after the end of treatment (EOT) for the clinical outcome. Deceased patients during the follow-up period were analyzed until the last provided data.

We recorded epidemiological data and baseline characteristics, including post-transplant complications and immunosuppressant medication. Clinical data regarding type of infection, previous antifungal treatment or admission in the intensive care unit were registered. After the inclusion of a patient, clinical and laboratory changes in blood count or biochemistry during the treatment were followed. Immunosuppressant dosing and blood levels including calcineurin (tacrolimus) and mTOR inhibitors were monitored during the isavuconazole treatment and after discontinuation. Dose modifications were guided by blood level determinations and were performed according to the attending physician. Pharmacological interactions were considered in case the dose of any immunosuppressive drug was modified at the time isavuconazole was administered. Renal failure, the administration of other drugs or any other known concomitant causes that lead to a change in immunosuppressive drugs dosing were ruled out. Lastly, any possible adverse event during the treatment was recorded. They were defined as new onset symptoms or laboratory changes after the initiation of isavuconazole, with improvement or disappearance after its withdrawal, and in the absence of other causes. Emergence of breakthrough FI during ISA treatment was also registered. ISA drug levels were not available.

### Definitions.

FI was defined by ISHLT consensus criteria (23) for lung transplant recipients and updated EORTC/MSGERC consensus definitions (24) for non-lung transplant recipients, taking into account specific considerations for invasive and noninvasive FI in SOT. Invasive FI was defined as clinical manifestations and microbiologic fungal isolation with demonstration of organ damage by radiology, bronchoscopy or biopsy, and excluding other causes. In noninvasive FI organ damage was not demonstrated (1). In lung transplant recipients, ulcerative tracheobronchitis was considered invasive FI, whereas simple tracheobronchitis and bronchial stent infection were classified as noninvasive fungal infection. Breakthrough FI was defined as an FI occurring during isavuconazole after 7 days of treatment or within 14 days of discontinuation (25). Clinical cure was defined as resolution of signs and symptoms of infection, along with the resolution of initial radiological and bronchoscopy (if it applies) changes at the EOT. Culture conversion was defined as the absence of the original pathogen in the same sample obtained at EOT. When this situation was not achieved it was considered as fungal persistence. Chronic lung allograft dysfunction (CLAD) was defined according to the ISHLT Pulmonary Council consensus report (26). Premature discontinuation was defined as ceasing of treatment by the treating clinician before completing the estimated duration of treatment. Suspected adverse events were graded according to CTCAE v5.0 (27). Hepatotoxicity was defined as the persistent elevation of cytolytic liver enzymes during treatment excluding other plausible causes, which was differentiated from isolated elevation of cholestatic liver enzymes above 2-fold the upper limit of normal.

### Microbiological data.

Isolates obtained from respiratory tract specimens and other locations were identified by MALDI-TOF (Vitek MS, bioMérieux) and assessed for antifungal susceptibility against Aspergillus spp. and fungal species. Susceptibility tests to amphotericin B, triazoles and echinocandins against molds were performed by diffusion with gradient strips (Etest). Microdilution was used in yeasts. All of them were interpreted according to the Clinical & Laboratory Standards Institute (CLSI) recommendations.

### Outcomes.

Primary outcome was the assessment of tolerability by means of occurrence of adverse events, treatment discontinuation and immunosuppressants dosing modification. Secondary outcomes were the clinical and microbiological response at the EOT, and the evidence of breakthrough FI during treatment with isavuconazole.

### Statistical analysis.

Quantitative variables are expressed as median and interquartile range (IQR). Categorical variables are expressed as numbers and percentages. Associations in dependent variables were tested using paired T-student’s test, and multivariate analysis was tested with analysis of variance (ANOVA) test. Significance tests were two-sided, and a *P* value of <0.05 was considered statistically significant. The statistical analysis was performed using SPSS Statistics version 23.0 (IBM SPP, Chicago, Illinois).

### Ethics.

The study was approved by the hospital ethics committee (VHI-ISA-2018-01) and the Spanish Drug Agency (approval OLE-ISA-2018-01).
