# Supplementary material for: Safety and Effectiveness of Isavuconazole Treatment for Fungal Infections in Solid Organ Transplant Recipients (ISASOT Study)
Source: Microbiol Spectr. 2022 Feb 16;10(1):e01784-21. doi: 10.1128/spectrum.01784-21 (PMC8849063; doi:10.1128/spectrum.01784-21)
Supplement: SUPPLEMENTAL FILE 1 — Supplemental material. Download SPECTRUM01784-21_Supp_1_seq3.pdf, PDF file, 0.1 MB [file spectrum01784-21_supp_1_seq3.pdf]

SUPPLEMENTARY MATERIAL

Supplementary table 1: Fungal isolates and secondary outcomes in lung transplant recipients and other SOTR.

	**Lung transplant recipients (n = 44)**	**Kidney, liver and combined transplant recipients (n = 9)**	**All SOTR (n = 53)**
**Identified fungal species**	**45 species in 35 lung transplant recipients**	**7 species in 9 kidney, liver and combined transplant recipients**	**53 species in 44 SOTR**
Aspergillus spp	36 (80)	7 (100)	43 (82.7)
A. flavus	14 (38.9)	2 (28.6)	16 (30.2)
A. terreus	11 (30.6)	0 (0)	11 (20.8)
A. fumigatus	8 (17.8)	4 (57.1)	12 (22.6)
Other Aspergillus spp	3 (6.7)	1 (14.3)	4 (7.6)
Mucormicosis	1 (2.2)	0 (0)	1 (1.9)
Other filamentous fungi	5 (11.1)	0 (0)	5 (9.4)
Yeasts	4 (8.9)	0 (0)	4 (7.5)
Trichosporon asahii	2 (4.5)	0 (0)	2 (3.8)
Candida albicans	1 (2.2)	0 (0)	1 (1.9)
Saccharomyces cerevisiae	1 (2.2)	0 (0)	1 (1.9)
**No fungal isolate**	**6**	**3**	**9 SOTR**
**Type of FI**[a]	**45 infections in 44 lung transplant recipients**[b]	**9 infections in 9 kidney, liver and combined transplant recipients**	**54 infections in 53 SOTR**[b]
Tracheobronchitis	22 (48.9)	3 (33.3)	25 (46.3)
Fungal pneumonia	7 (15.6)	0 (0)	7 (13)
Bronchial anastomotic infection	2 (4.4)	0 (0)	2 (3.7)
Other:			
Micetoma	5 (11.1)	1 (11.1)	6 (11.1)
Cutaneous infection	3 (6.7)	0 (0)	3 (5.6)
Disseminated FI	1 (2.2)	1 (11.1)	2 (3.7)
Osteomyelitis	1 (2.2)	0 (0)	1 (1.9)
Chronic otitis media	0 (0)	1 (11.1)	1 (1.9)

Isolation in donor	1 (2.2)	0 (0)	1 (1.9)
No proven or probable FI	3 (6.7)	3 (33.3)	6 (11.1)
**Clinical cure at EOT**	22 (50)	5 (55.6)	27 (50.9)
**Culture conversion in patients with fungal species isolation (n = 44)**	**35 lung transplant recipients**	**9 kidney, liver and combined transplant recipients**	**44 SOTR**
Culture conversion at EOT	14 (40)	3 (33.3)	17 (38.6)
Fungal persistence at EOT	14 (40)	1 (11.1)	15 (34.1)
Cultures were not performed at EOT	7 (20)	5 (55.6)	12 (27.3)

[a]All cases were definite or probable fungal infection according to ISHLT and EORTC/MSGERC criteria except when indicated.

[b]One patient was treated for fungal tracheobronchitis and subcutaneous infection at the same time.

Data are expressed as numbers (%) unless otherwise indicated. EORTC/MSGERC, European Organization for Research and Treatment of Cancer and the Mycoses Study Group Education and Research Consortium; FI, fungal infection; ISHLT, International Society for Heart and Lung Transplantation; SOTR, solid organ transplant recipient.

Supplementary table 2: *Aspergillus* spp MIC and susceptibility rates to the antifungal agents tested (n = 43).

		A. flavus (n =16, 37.2%. Fifteen MIC could be assessed)	*A. fumigatus* (n =12 , 27.9%. Ten MIC could be assessed)	*A. terreus* (n =11, 25.6%. Ten MIC could be assessed)	*A. nidulans* (2), *A. niger* (1), *A. lentulus* (1)
Isavuconazole	MIC50	0.25	0.19	0.38	0.125
	MIC90	0.38	0.38	0.5	NC
	IQR MIC50	0.125-0.38	0.125-0.25	0.19-0.5	0.047-051
Voriconazole	MIC50	0.25	0.125	0.38	0.125
	MIC90	0.5	0.25	1.5	NC
	IQR MIC50	0,19-0.38	0.125-0.25	0.25-0.5	0.094-0.19
Posaconazole	MIC50	0.25	0.125	0.094	0.38
	MIC90	0.5	0.38	0.38	NC
	IQR MIC50	0.19-0.38	0.064-0.25	0.064-0.25	0.19-0.5
Amphotericin B	MIC50	4	0.38	2	1
	MIC90	12	2	30	NC
	IQR MIC50	1.5-6	0.025-1	1.5-8	0.38-4
Anidulafungin	MIC50	0.002	0.008	0.008	0.016
	MIC90	0.006	0.047	0.03	NC
	IQR MIC50	0.002-0.004	0.003-0.03	0.006-0.16	0.004-0.064
Micafungin	MIC50	0.003	0.012	0.007	0.008
	MIC90	0.006	0.06	0.012	NC
	IQR MIC50	0.003-0.004	0.006-0.016	0.004-0.008	0.006-0.047

Caspofungin	MIC50	0.032	0.125	0.094	0.094
	MIC90	0.125	0.19	0.25	NC
	IQR MIC50	0.016-0.064	0.064-0.125	0.032-0.125	0.064-0.125

Data are expressed as numbers (%) unless otherwise indicated. IQR, interquartile range; MIC (μg/dL), Minimum Inhibitory Concentration (MIC50 and MIC90 values were defined as the lowest concentration of the antifungal at which 50 and 90% of the isolates were inhibited); NC, Not Calculated; S%, susceptibility percentage of isolates according to antifungal breakpoints established by CLSI.

Supplementary table 3: non *Aspergillus* species MIC to antifungal agents tested (n = 10)

	ISA	VOR	POS	AMPB	ANID	MICA	CASP
Candida albicans		0.008		1	0.015	0.125	0.125
Trichosporon asahii	0.094	0.06	0.25	0.5	32	32	32
Lomentospora prolificans	32	32	32	32	32	32	32
Alternaria alternata	8	8	8	2	0.03	0.06	0.5
Purpureocillium lilacinus	0.25	0.125	0.38	32	32	32	32
Trichosporon asahii, Saccharomyces cerevisiae, Mucor spp, *Diaporthe* spp, and unidentified new mold species	Antifungal susceptibility could not be performed						

MIC (μg/dL) = minimum inhibitory concentration.

Supplementary table 4: Dosage and plasmatic levels of tacrolimus and m-TOR inhibitors after starting isavuconazole

	Before start	1-2 days	4 days	7 days	10 days	14 days
Tacrolimus dose (mg)	4 (2-6)	3 (2-5.3)	2.3 (1.4-4)	2 (1.1-4)	2 (1.3-4)	2 (1-4)
	n = 47	n = 38	n = 46	n = 40	n = 37	n = 37
Tacrolimus measured levels	8.6 (5.7-11.4)	7.4 (4.8-15.2)	9.8 (5.7-15.2)	9.2 (4.8-10.9)	7.9 (6.1-9.4)	7.5 (5.6-10)
	n = 47	n = 28	n = 34	n = 24	n = 27	n = 28
mTOR dose	1.75 (1-2)	1 (1-2)	1 (0-1)	0.5 (0-1)	0.5 (0-1)	0.5 (0-1)
	n = 7	n = 7	n = 7	n = 7	n = 7	n = 7
mTOR measured levels	5.2 (2.4-7.4)	4.2 (3-9)	11.6 (7.1-12.6)	5.7 (3-9.4)	8.6	6.7 (4.4-7.3)
	n = 7	n = 4	n = 3	n = 4	n = 2	n = 4

Data are expressed as median (IQR) unless otherwise indicated.

Supplementary table 5: Dosage and plasmatic levels of tacrolimus and m-TOR inhibitors after discontinuation of isavuconazole.

	Initial	1-2 days	4 days	7 days	10 days	14 days
Tacrolimus dose (mg)	3 (2-4)	4 (2-4.9)	4 (2-4)	4 (2-6)	4 (2-5.3)	4 (2-6)
	n = 39	n = 30	n = 31	n = 31	n = 30	n = 30
Tacrolimus measured levels	6.8 (5.1-10)	7.9 (5.3-12.1)	9.3 (5.2-13.6)	6.7 (4.7-12.6)	7.3 (5.2-8.5)	7.2 (5.2-10.6)
	n = 39	n = 14	n = 17	n = 19	n = 15	n = 22
mTOR dose (mg)	1 (0.5-2.8)	1.5 (0.8-3.5)	1.5 (0.8-3.5)	1.5 (0.8-3.5)	1.5 (0.8-3.5)	1.5 (0.8-3.5)
	n = 5	n = 5	n = 5	n = 5	n = 5	n = 5
mTOR measured levels	5.7 (2.7-7.6)	7	5.1 (0.8-10.7)	3	1.9	3.9
	n = 4	n = 1	n = 3	n = 2	n = 2	n = 2

Data are expressed as median (IQR) unless otherwise indicated.

Supplementary table 6: Evolution of SOTR treated with ISA

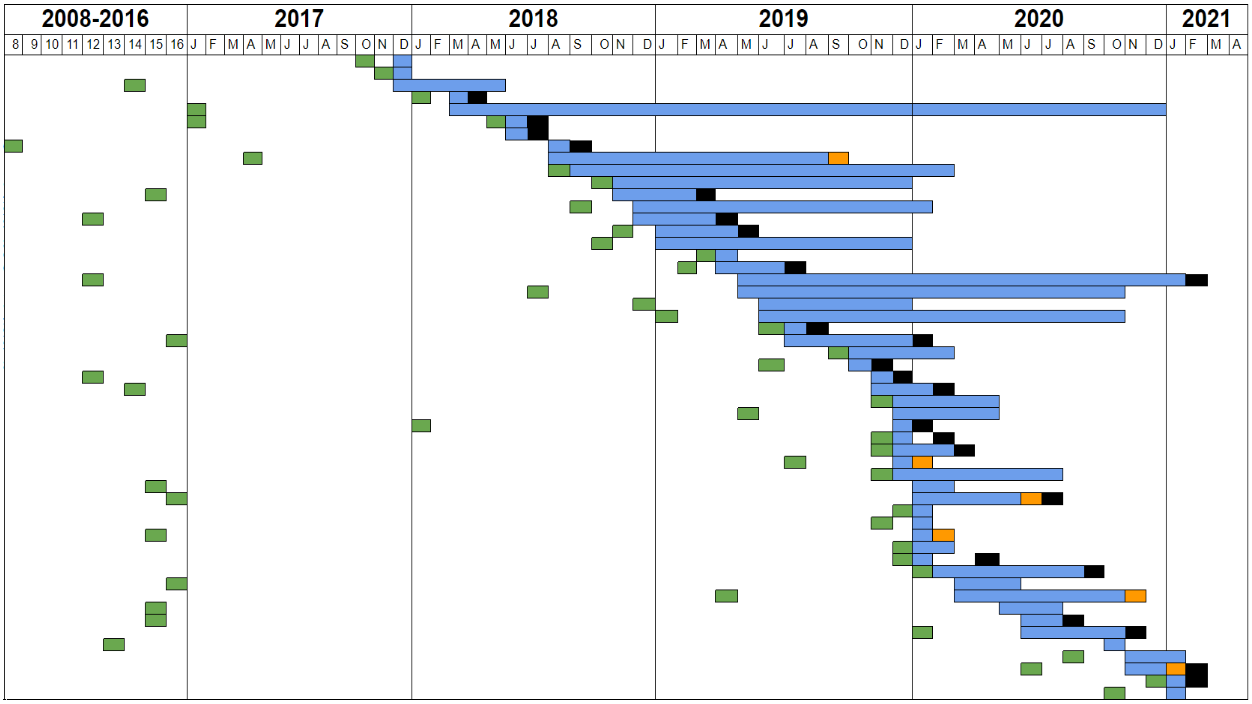

Black, deceased; Blue, duration of treatment with isavuconazole; Green, transplant date; Orange, discontinuation of treatment due to a clínically relevant adverse event. ISA, isavuconazole; SOTR, solid organ transplant recipient.
